# Phyto-assisted synthesized CuO NPs embedded in crosslinked zein/alginate composite films for hastening wound healing and tissue regeneration in rats bio-template

**DOI:** 10.1007/s13346-025-01948-z

**Published:** 2025-08-29

**Authors:** Sarah A. El-Lakany, Nazik A. Elgindy, Elbadawy A. Kamoun, Perusi M. Masanga, Shahira H. EL-Moslamy, Marwa Abu-Serie, Rania G. Aly, Noha Khalifa Abo Aasy

**Affiliations:** 1 Department of Industrial Pharmacy, Faculty of Pharmacy, Alexandria University, 1 El Khartoum Square, PO Box 21521, Alexandria, Egypt; 2 College of Pharmacy, Arab Academy for Science, Technology & Maritime Transport, Alexandria, Egypt; 3 Department of Chemistry, College of Science, King Faisal University, Al-Ahsa, 31982 Saudi Arabia; 4 Polymeric Materials Research Department, Advanced Technology and New Materials Research Institute (ATNMRI), City of Scientific Research and Technological Applications (SRTA-City), New Borg Al-Arab City, Alexandria, 21934 Egypt; 5 ST.Johns University of Tanzania, Dodoma, Tanzania; 6 Bioprocess Development Department (BID), Genetic Engineering and Biotechnology Research Institute (GEBRI), City of Scientific Research and Technological Applications (SRTA-City), New Borg Al-Arab City, Alexandria, 21934 Egypt; 7 Medical Biotechnology Department (MBD), Genetic Engineering and Biotechnology Research Institute (GEBRI), City of Scientific Research and Technological Applications (SRTA-City), New Borg Al-Arab City, Alexandria, 21934 Egypt; 8 Department of Surgical Pathology, Faculty of Medicine, Alexandria University, Alexandria, Egypt

**Keywords:** Zein, Alginate, Composite films, CuO, Tissue engineering, Wound healing

## Abstract

Biopolymer-based composite films were primed by incorporating alginate and zein natural polymers using a solution-casting method and superbly assisted by eco-friendly prepared copper oxide nanoparticles (CuO NPs). The influence of the addition method of CaCl_2_ as a crosslinker and CuO NPs loading content (0.1, 0.2, and 0.4 wt%) on the microstructural, physical, and mechanical properties of the films, were appraised. The formation of composite films and incorporation of CuO NPs were verified by FT-IR and XRD studies. The results unearthed that double crosslinking (Dipping method) succeeded in forming a firm, homogenous film that maintains its integrity in water for up to 24 h in comparison to the single (Blending) method. Inclusion of zein in the film and further loading with CuO NPs are manifested in a significant decrease in water vapor permeability, swelling and degradation percentage about (58.57, 52.26, and 25.80%); respectively. In addition to 1.26-folds increase in the tensile strength and 1.19-folds decrease in elongation to break, endorsing the excellent barrier property and durability of the formed films. Nevertheless, CuO loaded composite film proposes high biocompatibility against HBF4 cells with the highest IC50 and EC100 values, compared to alginate film and free-CuO NPs. The composite film exhibited the most effective antimicrobial activity against extremely drug-resistant human pathogens of both *Gram*-ve and *Gram* + ve bacteria strains, as well as fungal cells. The healed diabetic wound demonstrated an intact fully thickened keratinized epidermal epithelialization, and a complete absence of any inflammatory infiltrate after 13 days of treatment, emphasizing its suitability as a promising dressing candidate for skin tissue bioengineering.

**Graphical Abstract:**

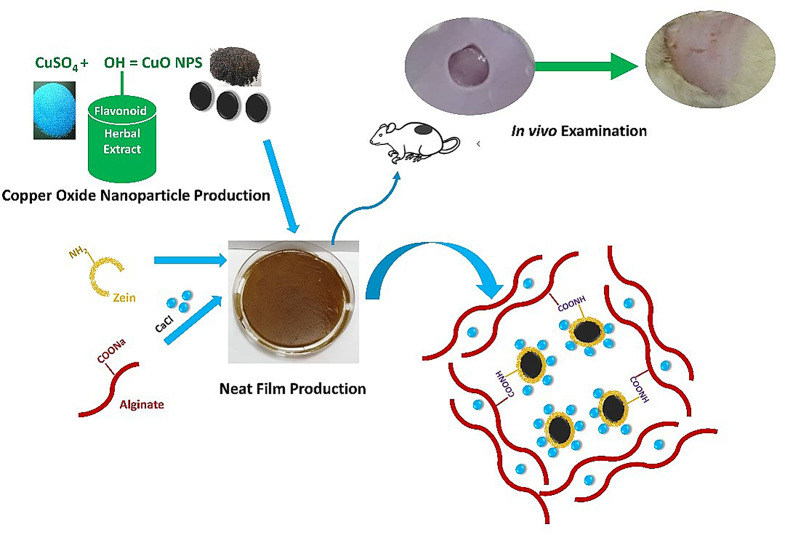

## Introduction

The skin is the main protective organ of humans and animals blocking the influx of harmful foreign bodies and setting the temperature, water, and electrolytes at balance [1]. Naturally, skin injuries heal by restoring the damaged epidermis and/or dermis layers through the subsequent healing activities including fibrin clot formation, inflammatory cytokines release and tissue growth, maturation, and differentiation [2]. Under certain pathological circumstances, an imbalance between the proliferation, remodeling, and vascularization stages occurs leading to defective healing pathways. The metabolic disease diabetes, manifested by high glucose levels in the blood, is considered a main cause of impairing natural wound healing via intervening with its various process activities. In fact, glucose binds firmly to Hemoglobin leading to damaging the vasculature system and thus insufficient transport of nutrients and oxygen to the wounded area and impairing the cells’ migration and proliferation processes [3]. The other cause of diabetic wound healing impairment is the synthesis of nitric oxide due to high sugar-blood levels generating reactive oxygen species (ROS). The prevalence of diabetic skin injuries is accompanied by undesired health and economic restraints on the health systems which leads to economic depletion, especially in developing countries.

A common approach towards creating a suitable healing environment for a diabetic wound is the application of a well-designed dressing on the skin which allows self-medication, bypassing hepatic first-pass effect, and minimalizing the systemic adverse effects. Different Topical formulations are used as dressings, these include polymeric materials that are formulated informs of films, nanofibers, and hydrogels. Polymeric dressings act as a shelter to prevent bacterial or fungal wound invasion, a support to minimize mechanical damage, and an absorbent for excessive exudate. The employment of biological polymers further improved the wound healing activity where these polymers are biocompatible and biodegradable materials with an effectively porous structure mimicking the extracellular matrix (ECM).

The anionic polysaccharide, alginate, extracted from brown seaweed plants, is currently used for plentiful biomedical applications. On one hand, this can be attributed to its relative safety, low cost, biocompatibility, biodegradability and abundance in nature. On the other hand, the main hindering factors to alginate use include its high-water solubility and tremendously fragile mechanical strength. To create alginate formulations with acceptable water resistance and enhanced mechanical properties, proper crosslinkers and/or nano-reinforcing fillers were added to such formulations [4]. It was found that a carbonyl group, present in the guluronate unit of alginate, can react with divalent ions to form a three dimensional crosslinked network with enhanced physical, chemical, and mechanical properties [5]. Thus, calcium cations were successfully used as crosslinking agents for alginate formulations in previous research works [6].

The corn protein polymer, zein, is a safe, biodegradable, biocompatible, low water vapor permeable and greaseproof material. Interestingly, around 40–80% of the composing amino acids of zein have a hydrophobic nature. Owing to such a hydrophobic property, zein is regarded as an alluring natural protein polymer to boost the biocompatibility of a variety of formulations where its hydrophobic nature allows the interaction with membrane receptors [7–10]. It also has a role as a primary carrier for the controlled drug release of hydrophobic active ingredients [11–13]. However, the zein-fabricated films suffer from poor elasticity and ductility. Such challenges were recently addressed by combining the hydrophobic zein with various polymers including, chitosan [14], polycaprolactone [15] and collagen [16]. These bio combinations create films with enhanced characteristics that can serve as a replacement for the biological tissue in the healing process.

Ecofriendly-synthesized copper oxide nanoparticles (CuO NPs) demonstrated unique characteristics that enabled their employment in various disciplines. Copper itself is a trace element essential acting as a crucial co-factor to produce numerous enzymes involved in the maintenance of the immunity responses. When copper occurs in the metal oxide form, it acquires metal ion attractive properties including being hydrophobic, highly porous, and a surface-charged moiety. The formulation of CuO in the nanoform leads to tailoring the obtained particle size of the NPs above 10 nm resulting in diminishing the reported harmful effect of 10 nm particles [17]. CuO NPs demonstrated reported potent bactericidal activity on diverse pathogens which can be compared to antibiotics in terms of efficiency [18–20].

The involvement of the green synthesis tactics succeeded in shrinking the toxicity of the developed CuO NPs for the living beings and their environmental hazards [18]. The produced bio-template is also remarkably stable, cheap and conveniently attainable compared to other metal oxides.

In the current study, green-synthesized CuO NPs were incorporated into zein/alginate composite film for speeding up the healing process of topical wounds in diabetes- induced male rats. CuO NPs were intended to enhance and accelerate the wound-healing rate, due to their biological actions. CuO NPs were favored as antibacterial agents, because they can overcome the problem of drug resistance encountered in conventional antibiotic usage. Zein protein polymer was chosen based on its safe nature alongside its adhesiveness to the wound area, whereas alginate polymers were also chosen based on their ability to absorb the huge amounts of the wound exudates to create a moist environment favorable during the healing process. To the best of our knowledge, this is the first successful attempt to incorporate CuO NPs into zein/alginate composite film for accelerating the wound healing process.

## Materials and methods

### Materials

#### Bacterial strains, plant material, and chemicals

Gram-positive bacterial cells including Streptococcus spp. ATCC 49619, Staphylococcus epidermidis ATCC 12228, and Staphylococcus aureus ATCC 6538), Gram-negative bacterial cells (Shigella spp. ATCC 11126, Escherichia coli ATCC 10536, Pseudomonas aeruginosa ATCC 27853, and Salmonella paratyphi ATCC 9150), in addition to fungal cells (Candida albicans ATCC 10231, Candida krusei ATCC 6258, and Saccharomyces cerevisiae ATCC 9763) were supplied by Bioprocess Development Department, GEBRI, SRTA-City, Alexandria, Egypt. Punica granatum L., Family Lythraceae peels powder was bought from El-Zaafarany Co. Egypt, (Batch number 6287000000313). Copper sulfate (CuSO4.5H2O) was purchased from Alpha Xhemika, India. Zein, poloxamer 407 (PLX), 3-(4,5-dimethylthiazol 2-yl) 2,5-diphenyl tetrazolium bromide (MTT), and sterilized Mueller Hinton broth were purchased from Sigma Aldrich Chemie, ST. Louis USA. Sodium alginate (medium viscosity) was purchased from (Lba Chemie, PVT, LTD, India. Calcium chloride (CaCL_2_) was purchased from Chem-Lab, Belgium. Sterile-filtered Dulbecco’s modified Eagle’s medium (DMEM) high glucose with L-glutamine was obtained from BioWhittaker (Lonza, Belgium). Normal human skin fibroblast (HBF4) cell line was got from the American Type Culture Collection (ATCC). CD45 (Ready-to-use primary antibody, mouse anti-human, monoclonal antibody, P0042) and α-SMA (Ready-to-use primary antibody, mouse anti- human, monoclonal antibody, P0943) were supplied by Leica Biosystems, USA.; Leica Biosystems, USA. Other used reagents and chemicals are of analytical grades..

#### Animals

Male albino rats (230 ± 20 g) were caged solely in an environment free of pathogen under standard conditions. All experimental procedures were performed in accordance with the approved protocol of the Animal Care and Use Committee of the Faculty of Pharmacy, Alexandria University, Egypt (Au: 06/2022/9/5/1/126) and the rules of the National Research Council’s guide for the care and use of laboratory animals. Furthermore, animal experiments followed the ARRIVE guidelines and the UK Animals (Scientific Procedures) Act, 1986, and associated guidelines, EU Directive 2010/63/EU for animal experiments.

### Methods

#### Green-synthesis of copper oxide nanoparticles (CuO NPs)

Copper oxide nanoparticles (CuO NPs) were synthesized following the experienced method performed by Aasy et al. [21]. In brief, *Punica granatum* extract was prepared by mixing 10 g of powdered plant in 100 mL of distilled water under magnetic stirring for 10 min at 55 °C. The mixture was then left to cool and filtered by Whatman No.1 filter paper. Copper sulfate solution was prepared with 15 mM of CuSO_4_.5H_2_O salt in 100 mL distilled water (containing 2 g of PLX). Subsequently, freshly prepared extract (90 mL) was mixed with 10 mL sulfate solution and left on a magnetic stirrer for 30 min at room temperature (RT). The mixture was then subjected to centrifugation at 8000 rpm for 10 min using Sigma Laboratory Refrigerated Centrifuge, Model 3 K-30, Germany, to separate the formed CuO NPs. The powder of CuO NPs was then washed with distilled water containing 50% v/v ethanol and left for air drying at RT.

#### Formulation and optimization of alginate (AFs) and alginate/zein films (AZFs)

##### Preparation of plain un-crosslinked AFs and AZFs (F1, F2)

A stock solution of 2% w/v alginate solution was prepared by using a magnetic stirrer at a temperature not exceeding 70^◦^C. While 2% w/v zein stock solution was prepared by dissolving zein portion-wise into an aqueous ethanolic solution (70% v/v). For the preparation of alginate films (AFs), alginate solutions were poured onto glass plates (8–9 cm) mounted with glycerol as a plasticizer. Films were allowed to dry at RT for about 24 h and then the dried films were blasted off from the plates, (F1). For the preparation of alginate/zein films (AZFs), aliquots from zein stock solution were mixed with alginate in a volume ratio of (1:2.5; zein: alginate) as a film-forming solution and treated as described above, (F2). The total volume of the mixtures was kept at 14 mL which controls the thickness of formed films.

##### Preparation of CaCl_2_ crosslinked AFs and AZFs (F3-F10)

For crosslinking AFs and AZFs with CaCl_2_, two different methods were employed namely blending and dipping methods. The first method, known as “blending method or single crosslinking method”, was based on the addition of 0.2% w/v CaCl_2_ directly into the film-forming solutions followed by the same preparation method as described above (F3 and F4). The second method, known as “dipping method or combined crosslinking method”, was based on immersing previously dried crosslinked AFs or AZFs in 5 mL of 5% w/v CaCl_2_ solution for 5 min, then the excess CaCl_2_ solution was casted-off by multi-washing, and the treated films were left to dry at RT for 6 h. (F5-F10) [22].

##### Preparation of CuO NPs-loaded AFs and AZFs

Different concentrations of CuO NPs (0.1, 0.2, and 0.4% w/v) were loaded into films. CuO NPs were dispersed in the film-forming solutions and left onto magnetic stirrer for 30 min to be dispersed efficiently. Then the mixtures were sonicated (Julabo sonicator, model USR3, Julabo Labortechnik, Ceelbach, Germany) for 5 min to ensure complete dispersion of CuO NPs before spreading on the plates. All prepared formulations are summarized in Table 1.

**Table 1:** Composition of films formulation and method of crosslinking

Formulae code	Alginate (%, w/v)	Zein (%, w/v)	CaCl_2_ (% w/v) (mixing method)	CaCl_2_ (% w/v) (immersion method)	CuO NPs (%, w/v)
F1	2	─	─	─	─
F2	2	2	─	─	─
F3	2	─	0.2	─	─
F4	2	2	0.2	─	─
F5	2	─	0.2	5	─
F6	2	2	0.2	5	─
F7	2		0.2	5	0.4
F8	2	2	0.2	5	0.1
F9	2	2	0.2	5	0.2
F10	2	2	0.2	5	0.4

* Alginate and zein were mixed in a volume ratio of (2.5:1)

### Characterization of film formulations

#### Film thickness

Film thickness was measured using a micrometer (Dial thickness gauge 7301, Mitutoyo Co., Japan). An Average of five measurements were taken in thickness calculations for each film, four around the perimeter, and one at the center.

#### FT-IR analysis

FT-IR spectra of film formulations (F5-F10) were recorded by FT-IR spectrophotometer model (Perkin-Elmer instruments, USA) at a resolution of 1 cm^− 1^ and a scanning range of 4000 to 400 cm^− 1^.

#### SEM investigation

A scanning electron microscope, SEM model (JOEL-JSM-6510, Japan) operating under vacuum and voltage of 5 kV was exploited for imaging the fractured and gold-coated surface of film formulations (F5-F10).

#### X-ray diffraction (XRD)

X-ray diffraction (XRD) patterns of F5, F6, and F10 films were attained using a powder X-ray diffractometer (Philps X’Pert Pro X-ray diffractometer) with Cu Ka radiations (k = 1.54060 nm) in a 2 h range to guarantee Cu NPs purity.

#### Moisture uptake (MU%)

Precise slices of all films were cut (3 cm^2^), weighed (W_0_) and then placed solely on *Petri* dishes in a desiccator filled with water. Swelled slices were removed after 24 h, dried with filter papers and then re-weighed (Ws). The subsequent Eq. 1 was applied to calculate MU% [9].

1 $$\:\%\:MU\:=\:(W_s-W_0)\:*100\:/W_0$$

#### Swelling and degradation percentage (SW% and D%)

*Swelling and degradation percentages (SW% and D%)* were determined to evaluate the influence of CaCl_2_ treatment on the resilience of the prepared films (F1-F10). Precise specimens ( 3 cm^2^) of films were weighed individually (W1) and immersed in a glass petri dish containing distilled water. Specimens were isolated after (1, 3, 5, and 24 h). At each time interval, excess water was dried by filter paper and subsequently swollen films were re-weighed (W2). Changes in samples’ weight and SW% were calculated, as directed in Eq. 2. Films were then left for complete air drying (24 h), and the dry un-solubilized remains were reweighed (W3). The weight of degraded film parts was calculated using the subsequent Eq. 3 [22].

2 $$\:SW\%=\:(W_2-W_1)\:*100/W_1$$

3 $$\:D\%=\:(W_1-W_3)\:*100/W_1$$

#### Mechanical properties

A standard uniaxial tensile test (Z050, Zwick Roell AG, Ulm, Germany) was utilized to evaluate the tensile strength (N/mm^2^), elongation-to-break (%E), and the elastic modulus (N/ mm^2^) of rectangular pieces (40 × 10 mm) of dried films (F5-F10). In brief, films pieces were attached to paper frames subjected to an elongation speed of 10 mm/min and 50 N cell loads. Each film was measured at sextuplicate to obtain an average reading ± SD [10, 23]. 

### Antimicrobial activity of film by disc and well-diffusion methods

#### In vitro antagonistic effect of film formulations

##### Antimicrobial susceptibility tests

Tests were performed for human pathogens using different antibiotic dices such as Kanamycin (K-30 mcg), Ampicillin (AMP-10mcg), Tetracycline (TE-30mcg), and Penicillin G (P-10U) for bacterial cells. Itraconazole (IT- 10mcg), Clotrimazole (CC-10 mcg), Ketoconazole (KT-30 mcg) and Fluconazole (FLC-25 mcg) were used for fungal cells using the disc diffusion assay [24–26].

##### The antagonistic potential

AZFs containing different concentrations of CuO NPs such as 0.1%w/v, F8 (IV), 0.2%w/v, F9 (V), and 0.4%w/v, F10 (VI) were assessed and compared with films that contained only Alginate, F5 (I), Alginate-0.4% CuO NPs, F7 (II), and plain Alginate-Zein, F6 (III) via disc diffusion method [27]. Before that, the liquid phases of alginate/zein/CuO NPs were prepared to assess the antimicrobial activities using well-diffusion procedure [28, 29].

##### Pre-cultures preparation

Tested human pathogens were inoculated separately in sterilized Mueller Hinton broth (17.5 g/L acid hydrolysate of casein, 2.0 g/L beef extract, and 1.5 g/L starch) and cultivated for 24 h at 37 °C and 200 rpm. These pre-cultures were then swabbed over the surface of solid LB plates (150 mm), and then NPs discs (6 mm) were placed on the top of them. However, wells were bored using metal corn borers (6 mm), and 100 µL of different tested NPs were loaded. Subsequently, the diffusion took place at 4 °C for 3 h, then these plates were incubated at 30 °C for fungal cells and 37 °C of bacterial cells for 48 h [30].

### In-vitro cell viability (%)

Normal human skin fibroblast HBF4 cells were grown in a 37 °C in DMEM medium containing 10% fetal bovine serum (FBS) in a 96-well cell culture plate for 24 h. Cells were used up to detect the cytotoxicity of CuO NPs loaded-AFs (F7) and AZFs (F10); in addition to free CuO NPs using MTT reduction method. The effective doses of tested formulations at 50% and 100% cell viability (IC_50_ and EC_100_), were also measured by the *GraphPad InStat* software [21].

### In vivo wound healing activity of CuO NPs and film formulations

#### In vivo excision wounds in diabetic rats

Primarily, diabetes is induced in rats as previously reported [21], where rats administered an intraperitoneal (I.P.) dose of streptozotocin (60 mg/kg) and blood-glucose levels exceeding 300 mg dL^− 1^ was considered an indication of a diabetic rat model. Eventually, after 14 days, an I.P. dose of pentobarbital sodium solution (1 mg/kg) was taken to hypnotize the rats and rounded-skin wounds (1 cm^2^ full-thickness) were developed after shaving the dorsum skin cautiously and sterilizing using a solution of cetrimide-chlorhexidine (1:30) and ethanol (70%) [21, 31].

Rats were then divided into five groups (6 rats each): untreated positive controls, blank AZF (F6), CuO powder (10 mg/cm^2^), CuO-AF (F7), and CuO-AZF (F10) treated groups. On the subsequent days (0, 3, 7, and 10), the films were topically applied to the wound regions of all film-treated groups and sheltered with a porous 3 M Micropore TM adhesive tape. The wound regions were pictured and the percentage of wound closure (WC%) as well as wound half closure time (WC_50_) were assessed at the predetermined time intervals as described by Parasad and Dorle [32]. On day 13, rats were sacrificed, and wound regions were excised for histological and immune-histochemical examination [33]. 

#### Histological immunohistochemical (IHC) staining examination

To evaluate the wounds’ histological features and healing process, the wound tissue specimens were stained with Hematoxylin and eosin (H&E) and Masson-Trichrome special staining (MT). *ImageJ*, *v1.53* (Maryland, USA) was utilized to approximate the fibrous tissue, granulation tissue, and epidermal thickness percentages. Moreover, the skin appendages were totaled (no skin appendages scored 1, few appendages < 5/wound areas scored 2, while ≥ 5 per wound areas scored 3). Skin sections were subjected to an IHC staining using CD45 and α-SMA to stain the lymphocytes, blood vessels, and myofibroblasts, respectively. The quantitative image analysis (Leica Microsystems, Switzerland) was adapted for IHC quantification [21, 31].

### Statistical analysis

All measurements were carried out at least in triplicate. Data are displayed as mean ± standard deviation (SD). *ANOVA* and *Tu*key multiple comparisons tests were used to compare the results of each group statistically using Minitab 18 software (*Minitab*^®^ *18.1*,* ©2017*) using a one-way analysis variance (*P*-value ≤ 0.05).

## Results and discussion

### Green-synthesis of copper oxide nanoparticles (CuO NPs)

The greenly synthesized CuO NPs were successfully prepared and fully characterized in our previous published work [21]. SEM results showed spherically shaped particles with around 230 nm in diameter.

### Film formation and optimization

**Fig. 1:**
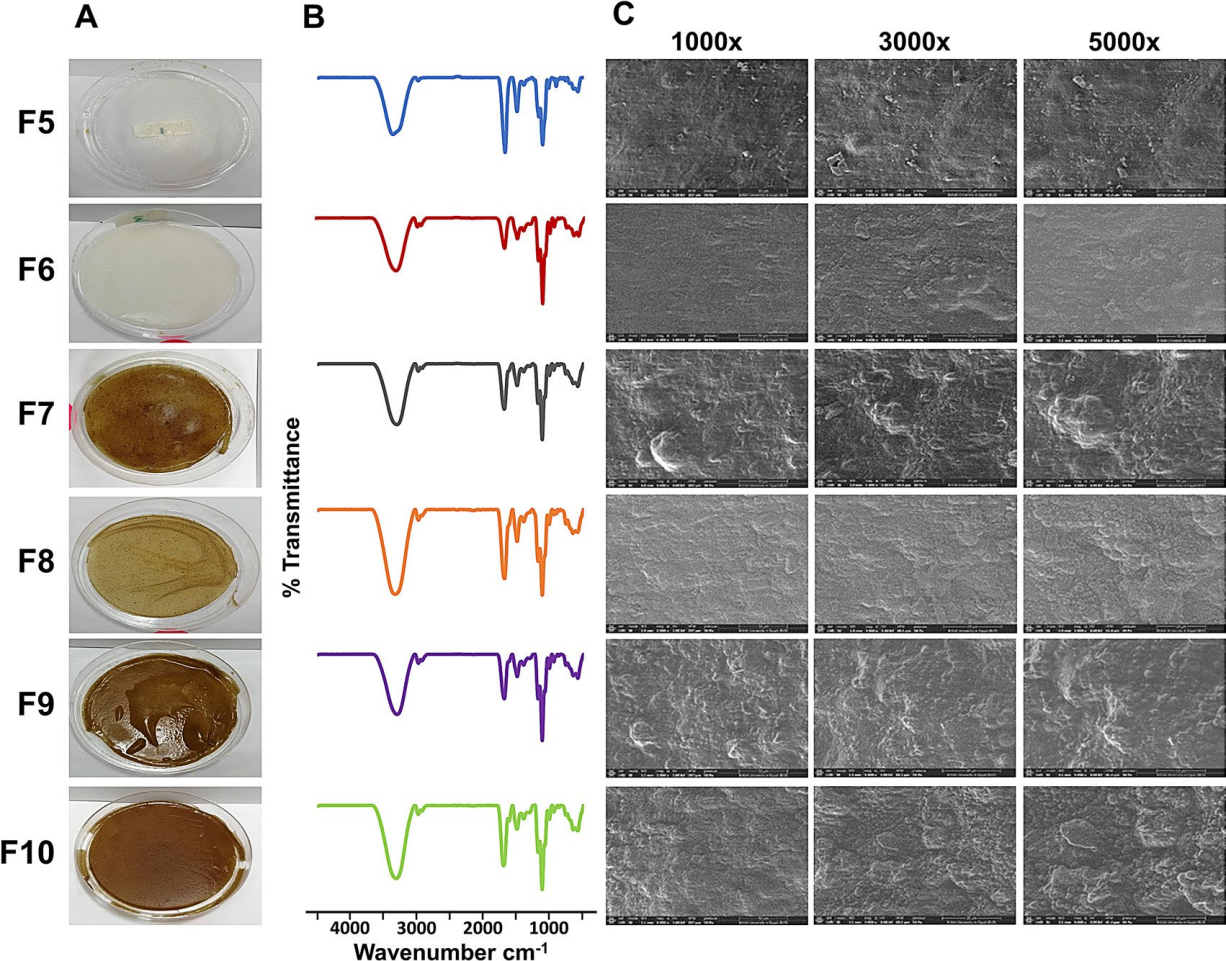
(**A**) Photographed images, (**B**) FTIR patterns and (**C**) SEM images of blank AF (F5), blank AZF (F6), 0.4% w/v CuO NPs-loaded AF (F7), 0.1% w/v CuO NPs-loaded AZF (F8), 0.2% w/v CuO NPs-loaded AZF (F9), and 0.4% w/v CuO NPs-loaded AZF (F10)

Simple visual inspection of plain AFs and AZFs prepared with/without CaCl_2_ treatment (control films) indicates that all films are flexible, transparent, homogenous, with no evidence of air bubbles or agglomerations and pliable (Fig. 1A). While the yellow shade of loaded films comes mainly from the intrinsic color of CuO powder (Fig. 1A). In the preparation of the ‘blinding films’, CaCl_2_ solution was mixed directly into alginate solution and then casted into films.

In the second *dipping* method, blinding films were then soaked in 5%, w/v CaCl_2_ solution for 5 min. After soaking, the films were found to dry much faster than the control or blending films, resulting in slightly translucent, stiff films, referred to as “dipping films”. The quality of the control and blinding films was not good, and they can readily degrade totally in an aqueous medium within 5 min. This may be credited to the hydrophilic nature of alginate, as well as possibly to the inadequate gelation with a small amount of Ca^2+^ elicited partial crosslinking and faster hydrolysis. Conversely, better film quality was attained from the dipping films; this may be appertaining to the crosslinking of alginate molecules with sufficient (Ca^2+^) molecules, producing water-insoluble crosslinked calcium alginate films by the ion exchange reaction which, in turn, facilitated their subsequent fabrication into dimensionally firm films. Dipping treatment enriched the mechanical integrity of the “blinding” films [6, 34]. Dipping films preparation method was chosen as the optimized formulation for further CuO NPs loading and evaluation.

### Characterization of the tailored films

#### Film thickness

The film thickness was significantly dependent on the film composition and their preparation method (Table 2). It was observed that the thickness of the ‘blending films’ is recorded of (179–196 mm), followed by the control (169–189 mm) and then the ‘dipping films” (168–188 mm). The thickness of the dipping films seems to decrease as some alginate molecules were crosslinked by Ca^2+^ which occurred at the surface of the films, resulting in more packed molecules with a reduction in thickness [6]. However, the incorporation of zein into AFs interprets a significant (P < 0.05) thickness increase in all methods (F2, F4, F6); compared to the plain AFs. This behavior may be associated with the differences in the molecular masses of the biopolymers. It was previously reported that zein films were thicker than alginate films constituting the same concentrations and volumes [35]. The thickness of the films did not significantly change with the presence of CuO NPs (P > 0.050).

**Table 2:** Study of water permability, swelling ratio (%) and degradation (%) of films coded (F1-F10)

Formulae code	Moisture uptake (%, w/v)	Swelling (%, w/v)	Degradation (%, w/v)	Film thickness (mm) ± SD
F1	598.45±10.32	─	100	0.169 *± 0.003*
F2	166.98±5.90	─	100	0.189 *± 0.002*
F3	391.24±2.12	─	100	0.179 *± 0.004*
F4	150.41±4.57	─	100	0.196 *± 0.005*
F5	168.22±3.44	1260±3.71	89.48±4.61	0.168 *± 0.003*
F6	133.05±9.07	914.51±2.90	69.75±1.98	0.188 *± 0.003*
F7	151.57±3.23	547.82±5.09	75.35±0.44	0.166 *± 0.004*
F8	118.46±5.49	902.59±3.78	68.67±3.72	0.187 *± 0.003*
F9	105.06±6.99	452.85±1.23	69.23±6.83	0.187 *± 0.001*
F10	55.10±8.87	436.58±0.65	66.39±5.13	0.188 *± 0.001*

#### FT-IR analysis

The spectrum of blank alginate film (F5) shows two peaks at ν 3334, and 2925 cm^− 1^ assigned to stretching vibrations of OH, and CH bonds: respectively. The peaks at ν 1086 and 1027 cm^− 1^ are attributed to the stretching vibration of C-O-C bond (Fig. 1B). The asymmetric COO^−^ vibrational peak shifted from ν 1595 to 1605 cm^− 1^, while the symmetric COO^−^ peak shifted to a higher wavenumber (from ν 1408 to 1422 cm^− 1^). This can be accredited to the interaction between carboxylic group and calcium ions, because of the crosslinking [34, 36].

Upon mixing zein with alginate, (F6), a peak band at ν 2960.1 cm^− 1^ almost appears and relates to the stretching vibration mode of a symmetric alkyl C–H C-H. Zein, as previously reported, shows two prominent absorption peaks at ν 1690 and 1550 cm^− 1^ typical amide I and II bands. The amide I band gradually shifts from ν 1690 to 1618.5 cm^− 1^ with decreased intensity. While ν 1550 cm^− 1^ appears at the same wavenumber [37]. Moreover, a long broad peak band at ν 3334 cm^− 1^ is attributed to O-H stretching vibration and was shifted to ν 3275 cm^− 1^. The result may indicate that hydrogen bonds between amide groups of glutamines in zein and hydroxyl groups in alginates are formed. The same results were previously obtained with a zein/propylene glycol alginate binary mixture (Fig. 1B) [38].

CuO NPs loaded films (F7-F10) are characterized by increased intensity of absorption peaks in the range of ν 500–700 cm^− 1^, which reveal the vibrational modes of Cu-O and confirm CuO NPs incorporation. In addition to, its characteristic peaks at ν 1335.06 cm^− 1^, 1260.94 cm^− 1^, 3389.82 and 2926 cm^− 1^, which relate to the stretching of COOH and amino groups absorption peaks, stretching of O-H groups of alcohols and phenols, N-H amines of the amides, in addition to C-H stretching of alkanes, that may be attached to the surface of CuO NPs; respectively, Fig. 1B [39].

#### SEM investigation

**Fig. 2:**
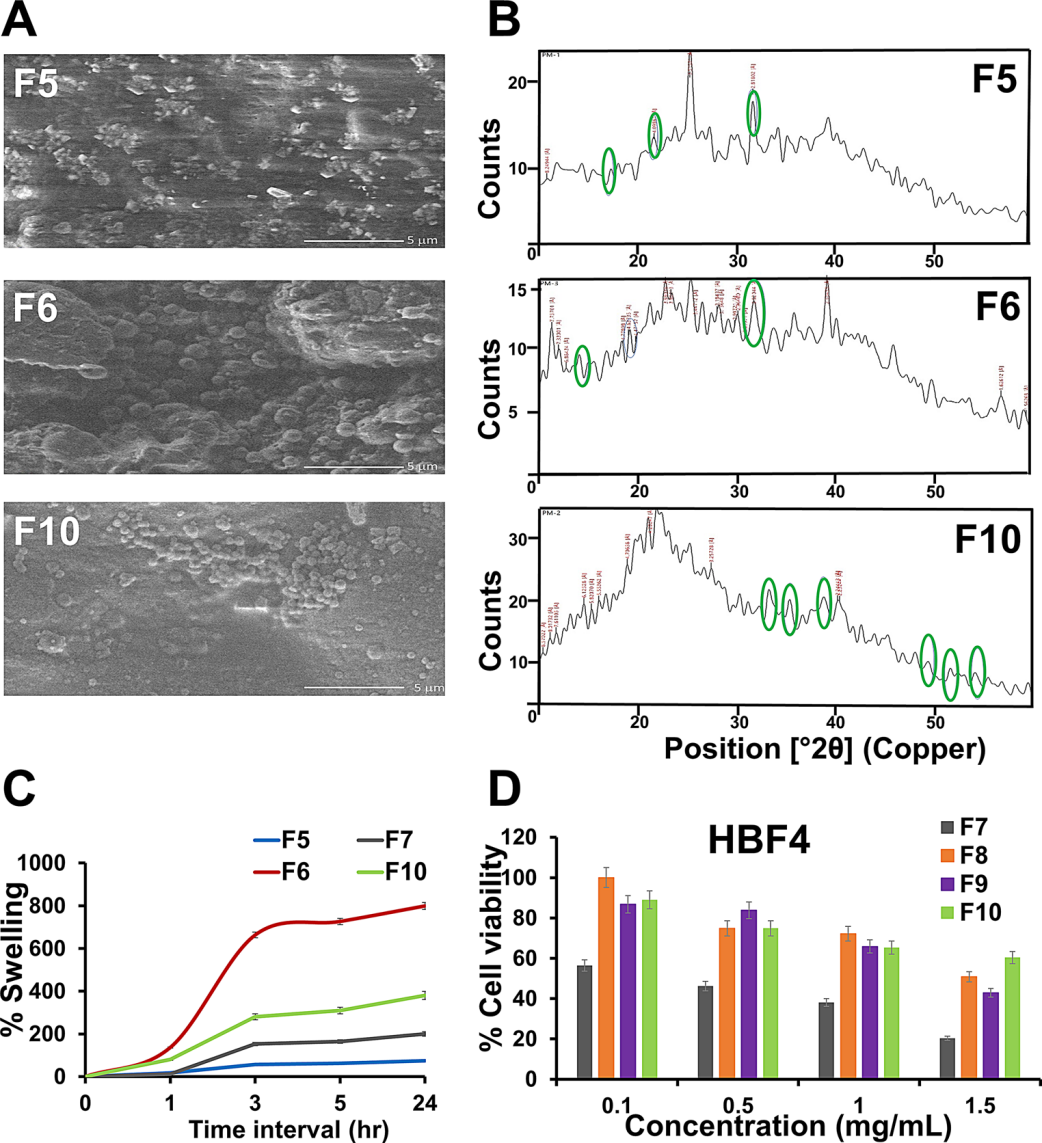
(**A**) SEM images of samples coded F5, F6, and F10 at high original magnification (10,000x), (**B**) XRD spectra of F5, F6, and F10; (**C**) swelling pattern of F5, F6, F7 and F10 in distilled water over 24 h at RT; and (**D**) % cell viability of plain CuO NPs powder, F7, and F10 at different concentrations (0-0.8 mg/mL) on HBF4 cell line

SEM images of crosslinked film formulations F5- F10 at different magnification powers are examined (Figs. 1C and 2). At high magnification, AFs (F5) crosslinked with Ca^2+^ are observed to be heterogeneous, and dense surface structural with large dents. Upon soaking the film in CaCl_2_, swelling and polymer folding occur resulting in a lack of surface homogeneity upon further drying (Fig. 2A) [34]. In accordance to Norajit et al. [40], a fibrous-like structure was observed when alginate was crosslinked with Ca^2+^ contrary to a homogenous plain uncross-linked alginate film. Interestingly, the addition of zein to alginate film, (F6), results in the formation of a particulate system, with a roughly spherical shape and a smaller diameter, adsorbed on the alginate matrix. This can be attributed to the antisolvent precipitation of zein; in addition, the presence of Ca^2+^ may take a part in crosslinking zein particles. The same finding is observed previously when zein is imaged in the presence and absence of Ca^2+^ and reported the formation of protein aggregates [38]. On the other hand, the incorporation of CuO in AZFs (F10) is evidenced by the appearance of small discrete crystals of various sizes dispersed throughout the mass of the films and appertaining to the fact that CuO is not water-soluble (Fig. 2A) [6].

#### X-ray diffraction (XRD)

Crosslinked alginate film (F5) exhibits two characteristic peaks at 2θ diffraction angles of 13° and 22°. Additional characteristic peaks for calcium alginate are observed at angles of 13°, 56°, 20.64°, 20.06°, 28.96° as well as 36.40°, revealing its semi-crystalline nature (Fig. 2B) [36]. Incorporation of zein onto alginate film (F6) reveals two broad peaks having maxima at 8.99◦ and 19.38◦ which are in an agreement with Kayaci and Uyar [41] and Sun et al. [38]. It is worth to mention that, compared to the separate patterns of zein and alginate, there is a distinctive new broad peak at the diffraction angles of 30° for zein-alginate bicomplex, which designating the formation of an amorphous complex with the intermolecular interaction occurred between both polymers. The same is observed by Sun et al. [38], when zein is combined with propylene glycol alginate. Meanwhile, the diffraction pattern of CuO NPs-loaded AZFs, F10, makes an evident that CuO maintains its crystalline nature. The diffraction angles are observed to be at 2θ ~ 32.47°, 35.49°, 38.68°, 48.65°, 53.36°, and 58.25° that are assigned to the reflection lines of monoclinic CuO NPs [42].

#### Moisture uptake (MU%)

Basically, both natural polymers can highly permeate and absorb water. AFs and AZFs are prepared by no or low concentration of CaCl_2_ as (blending films) show high MU% (Table 2). Crosslinked AFs (F3) shows a 34.62% reduction in MU%, compared to un-crosslinked AFs (F1). The same is observed in crosslinked AZFs (F4), where a 9.92% reduction is obtained, compared to un-crosslinked AZFs (F2).

Furthermore, it is observed that MU% of both Afs and AZFs films is affected greatly by the immersion in 5% CaCl_2_, dipping films (F5, F6). They show a significant reduction (*P* > 0.05) by 56.99 and 11.56%; respectively, compared to the corresponding blending films. It is assumed that ionic crosslinking imparted a reduced polymer segmental mobility especially at the surface; thus reducing MU% through the film matrix [6]. The addition of zein to all AFs shows a significant reduction (*P* < 0.05) in their MU%. This may be attributed to the hydrophobicity of zein creating hydrophobic spots within the hydrophilic matrix of alginate. In addition, it is suggested that water vapor transfer depends mainly on the ratio between the hydrophilic and hydrophobic portions as water vapor permeation occurs only through the hydrophilic portion [40].

Loading films with CuO NPs flaunts a reduction in MU% in all films and reduction increases as concentration of CuO NPs increases. AFs loaded with 0.4% w/v CuO NPs (F7) reveals a 16.7% reduction, compared to unloaded film (F5). While for loaded AZFs (F8-10) reduction of 10.9, 21.05, and 58.57% is obtained upon increasing CuO NPs from 0.1%, w/v to 0.2 and then to 0.4%, w/v. This may be attributed to the tortuous pathway created by impermeable crystalline CuO NPs distributed in the matrix. Thereby they increase the effective diffusion pass length travelled by the water molecules. The same has previously reported when AFs were loaded with cellulose NPs [4, 22]. Since it is recommended for the film to endure an excellent barrier property, and the low permeability of CuO NPs loaded films prove its suitability with enhanced barrier performance.

#### Swelling and degradation percentage (SW% and D%)

An effectual wound dressing must have the competence of absorbing wound exudates and gradually degrade at a suitable performance to allow a controllable drug release. Both control and blending films AFs and AZFs without CaCl_2_ treatment are almost utterly dissolved within 5 min after immersion in water, which makes it impossible to measure the SW% (Table 2). It may be attributed to the hydrophilic nature of alginate and no or inadequate crosslinking in the case of blending films. On the other hand, the dipping films retained their integrity for up to 24 h with increases SW% as the immersion time increases (Fig. 2C). The above phenomena is due to the crosslinking activity of CaCl_2,_ renders combined-crosslinked AFs and AZFs insoluble and resist the degradation in water [43].

It is observed that, encompassing zein in F6 film is accompanied by a significant decrease (*P* < 0.05) in SW and D% of films by 27.4 and 22.04%; respectively, compared to plain alginate film (F5). By taking into account the hydrophobic nature of zein incorporated in preparation of the films, the water degradation resistance increases [10]. It is also worth mentioning that, hydrogen bond formation between the two polymers improves the cohesiveness property of the biopolymer matrix and decreases water aptitude to break this bond [4].

To boot, loading of CuO NPs to films imparted an increase film durability with a significant reduction effect (*P* < 0.05) on both SW and D% [40]. AFs loaded with CuO NPs (F7) show a decrease in both 56.5% of SW% and 15.7% of D%, compared to unloaded film (F5). AZFs loaded with CuO NPs (F8-10) revealed CuO NPs concentration-dependent reduction in SW%. When CuO NPs increases from 0.1 (F8) to 0.4% (F10), the reduction in SW% increases from 1.36 to 52.26% with an insignificant influence on degradation (Table 2). This reduction in SW% of films may be related to a strong hydrogen bond formation between CuO NPs and the film matrix, attenuating water’s ability to break the bond; thus, SW% decreases [4]. Also, increasing CuO NPs concentration has insignificant (*P* > 0.05) influence on D%.

From the above results, it can be concluded that as the concentration of CuO NPs increases, the swelling percentages significantly decreases, which is an imperative characteristic that enables both drug release and absorption of excess exudate from the wound. The water absorption causes gradual degradation of the film membrane leading to the sustained release of active pharmaceutical ingredients.

#### Mechanical properties

Tensile strength (N/mm^2^), elongation to break (%E) and the elastic modulus (N/mm^2^) of crosslinked films are depicted in Table 3. The unloaded CaCl_2_-crosslinked film AF (F5), exhibits a high TS value of 35 ± 3.04 N/mm^2^, elastic modulus of 2.25 ± 0.19 N/mm^2^, and elongation percentage of 210 ± 4.73%; which can be correlated to the hydrocolloidal nature of alginate polymer that imparts flexibility and stoutness of alginate films [35]. The inclusion of zein in F6 decreases TS, elastic modulus, and elongation percentage values to 28 ± 1.71 N/mm^2^, 1.87 ± 0.32 N/mm^2^, and 171 ± 3.89%; respectively. An explanation for such a phenomenon is the fragility of zein that was previously reported [35]. In fact, zein films are biopolymer of protein origin with a brittle structure while alginate films have more elastic, well-organized, and strong film structures. The brittleness and elasticity disputes in zein films are sequels of strong hydrophobic reactions that stock zein molecules together; however, alginate could be used to reimburse these defects and recuperate its mechanical attributes [35]. 

**Table 3:** Mechanical properties of distinct films coded (F5-F10) (mean ± SD, *n* = 3)

Formulae code	Elongation to break (%) ± SD	Maximum tensile strength (*N*/mm^2^) ± SD	Stress/strain or Elastic modulus (*N*/mm ^2^) ± SD
F5	210 ± 4.73	35 ± 3.04	2.25 ± 0.19
F6	171 ± 3.89	28 ± 1.71	1.87 ± 0.32
F7	185 ± 2.52	49 ± 2.27	3.31 ± 0.28
F8	188 ± 3.75	44 ± 2.98	3.25 ± 0.35
F9	167 ± 4.15	49 ± 2.54	3.87 ± 0.23
F10	155 ± 1.93	62 ± 3.43	4.15 ± 0.21

It is observed that, the incorporation of CuO NPs (0.4%, w/v), F7, enhances the mechanical strength of AF. Moreover, increasing the concentrations of CuO NPs incorporated in AZFs results in a significant increase in the ultimate TS values. The TS values of F8, F9 and F10 are 44 ± 2.98, 49 ± 2.54 and 62 ± 3.43 N/mm^2^; respectively (Table 3). While the elastic modulus of films followed a similar trend to the TS. As the concentration of CuO NPs increases from 0.1 to 0.4%, w/v (F8-F10), the elastic modulus values increase from 3.25 ± 0.35 to 4.15 ± 0.21 N/mm^2^. This can be accredited to the formation of bonds between hydroxyl groups of the used polymers and CuO NPs. In accordance with previous research work, CuO NPs can play a role in enhancing the stiffness of AZFs by strong interfacial interaction across NPs-polymer matrix [44]. In general, the ultimate TS and elongation at break of biofilms are influenced by NPs distribution throughout the polymer matrix. It seems that the interaction between CuO NPs and the polymer matrix resulted in an increase in TS with reduced films ‘elongation at break, as a result of a decline in the polymer chains mobility. Similarly, in this work, a same decrease in the films’ elongation is observed as the concentration of CuO NPs increases, as shown in Table 3. The same findings was previously obtained where CuO NPs served as anti-plasticizing agents, that can diminish the flexibility of the films by enhancing the interactions and reducing the free spaces between the chains of the biopolymer [45, 46].

### Determination of antimicrobial activity of film formulations by disc and well-diffusion method

One of the most serious threats we face as a global community is antimicrobial resistance to common antibiotics [27, 28, 47]. As a result, discovering new antibacterial agents of alternative products with proficient properties in the treatment of these multi-drug resistant human pathogens is a top priority [30]. There have been several antibiotic resistance profiles discovered for human pathogens. As a result, terms including multidrug-resistant, extensively drug-resistant, and pan drug-resistant microbial cells were employed to characterize them [48]. Extensively multi-drug-resistant strains, according to the European Centre for Disease Prevention and Control, that are resistant to at least one antibiotic from three or more antimicrobial classes. On the other hand, multi-drug-resistant bacteria are only sensitive to one or two antibiotic classes, then pan drug-resistant microbial cells refer to microbes that are resistant to all commercially available or routinely tested antibiotics [25, 49]. So, in this work, the selected human pathogens were tested against several antibiotic classes, as indicated in Table 4. In susceptibility testing results, inhibitory zones were classified as having no activity (0 mm, ‘-‘), less activity (1–10 mm, ‘+’), moderately active (11–15 mm, ‘++’), and highly active (16–25 mm, ‘+++’). All examined human pathogens were classified as extensively multi-drug-resistance microbial cells because they were resistant to at least one of the antibiotics tested, as reported in Table 4.

**Table 4:** Antimicrobial susceptibility test results using different antibiotic categories (which films have been here tested)

Human pathogens	Antibiotic sensitivity discs			
	K-30 mcg	AMP-10 mcg	TE-30 µg	*P* 10 U
*Salmonella paratyphi*	-	-	-	++
*Shigella spp.*	-	+++	+	-
*Pseudomonas aeruginosa*	+	-	-	++
*Escherichia coli*	+	-	-	+
*Streptococcus spp.*	-	+++	-	+
*Staphylococcus epidermidis*	-	-	++	++
*Staphylococcus aureus*	-	++	-	++
	**IT-10 mcg**	**FLC − 25 mcg**	**CC-10 mcg**	**KT-30 mcg**
*Candida krusei*	-	-	-	++
*Saccharomyces cerevisiae*	+	++	-	-
*Candida albicans*	+	-	-	+

(+++): Inhibition zones (16–25 mm), (++): Inhibition zones (11–15 mm), +: Inhibition zones (1–10 mm), -: No inhibition zones

To improve metal oxide NPs activity, a variety of protein bases can be employed in the formulation of nano systems. Zein is one of the most frequently utilized protein tools for formulating nano-carriers in the food and pharmaceutical industries, due to its biodegradability, biocompatibility and low cost. Since zein can be used as an eco-friendly carrier system to provide acceptable release qualities and decrease toxicity in the environment [50–52]. Herein, zein with alginate are utilized for formulating different concentrations of CuO NPs such as 0.1%, F8 (IV), 0.2%, F9 (V), and 0.4%, F10 (VI). Then, the antagonistic effects of alginate/zein/CuO NPs films are assessed and compared to films that containing alginate, F5 (I), alginate/0.4% w/v CuO NPs, F7 (II), and alginate/zein, F6 (III) against extensively drug-resistant human pathogens using both disc and well diffusion methods (Table 5).

**Table 5:** Antagonistic efficacy of different films containing different concentrations of CuO nps; 0.1%w/v CuO NPs-loaded AZF, F8 (Treatment IV), 0.2%w/v CuO NPs-loaded AZF, F9 (Treatment V), and 0.4%w/v CuO NPs-loaded AZF, F10 (Treatment VI) were assessed and compared to films that contained only alginate, blank AF F5 (Treatment I), 0.4%w/v CuO NPs-loaded AF, F7 (Treatment II), and blank AZF, F6 (Treatment III) against multidrug-resistant human pathogens using both (D) disc and (W) well diffusion methods

Multidrug-resistant human pathogens	Zones of inhibition (mm ± SD)											
	Treatment I		Treatment II		Treatment III		Treatment IV		Treatment V		Treatment VI	
	D	W	D	W	D	W	D	W	D	W	D	W
*Salmonella paratyphi*	0.00	0.00	0.00	15.36 ± 4.23	0.00	0.00	0.00	10.38 ± 0.23	0.00	12.65 ± 2.56	14.23 ± 3.45	17.35 ± 5.46
*Shigella spp.*	0.00	0.00	12.36 ± 1.08	12.78 ± 6.56	0.00	0.00	0.00	13.89 ± 5.12	0.00	15.59 ± 3.45	15.45 ± 4.62	21.45 ± 4.69
*Pseudomonas aeruginosa*	0.00	0.00	8.48 ± 2.69	8.36 ± 1.96	0.00	0.00	0.00	14.08 ± 7.12	0.00	18.56 ± 3.56	18.36 ± 6.23	22.36 ± 3.89
Escherichia coli*	0.00	0.00	9.69 ± 5.78	23.89 ± 5.23	0.00	0.00	0.00	17.23 ± 4.65	0.00	15.69 ± 4.25	20.12 ± 4.56	25.78 ± 5.96
Streptococcus spp.*	0.00	0.00	0.00	12.78 ± 4.78	0.00	0.00	0.00	0.00	0.00	0.00	0.00	20.18 ± 3.69
*Staphylococcus epidermidis*	0.00	0.00	0.00	14.12 ± 3.56	0.00	0.00	0.00	0.00	0.00	13.72 ± 2.41	8.45 ± 4.89	19.7 ± 4.12
*Staphylococcus aureus*	0.00	0.00	0.00	17.09 ± 6.45	0.00	0.00	0.00	0.00	0.00	9.45 ± 3.56	0.00	18.96 ± 4.12
*Candida krusei*	0.00	0.00	0.00	12.98 ± 3.25	0.00	0.00	0.00	0.00	0.00	0.00	0.00	20.36 ± 2.59
Saccharomyces cerevisiae*	0.00	0.00	0.00	17.89 ± 2.45	0.00	0.00	0.00	0.00	0.00	8.68 ± 3.45	21.89 ± 3.59	23.79 ± 5.69
*Candida albicans*	0.00	0.00	5.89 ± 0.06	23.45 ± 5.36	0.00	0.00	0.00	12.35 ± 3.01	0.00	16.78 ± 4.56	17.56 ± 3.89	21.78 ± 4.69

**Fig. 3:**
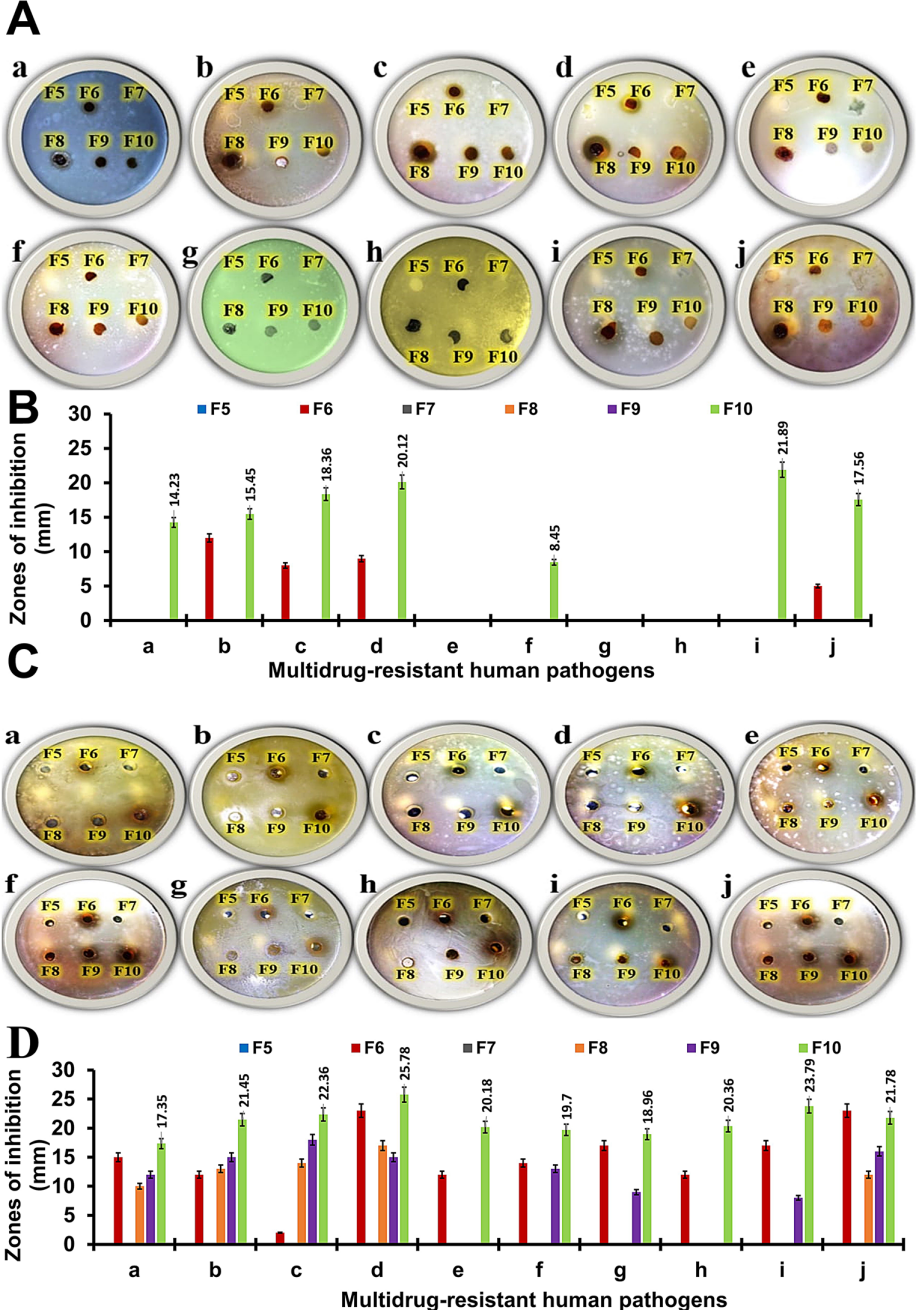
**(A)** Antimicrobial activity photographs showing different formed inhibition zone and **(B)** histogram showing the developed inhibition zone for blank AF (F5), blank AZF (F6), 0.4%w/v CuO NPs-loaded AF (F7), 0.1%w/v CuO NPs-loaded AZF (F8), 0.2%w/v CuO NPs-loaded AZF (F9), and 0.4%w/v CuO NPs-loaded AZF (F10); using disc-diffusion method against multidrug-resistant human pathogens (a) *Salmonella paratyphi*, (b) *Shigella spp.*, (c) *Pseudomonas aeruginosa*, (d) *Escherichia coli*, (e) *Streptococcus spp.*, (f) *Staphylococcus epidermidis*, (g) *Staphylococcus aureus*, (h) *Candida kruisei*, (i) *Saccharomyces cerevisiae* and (j) *Candida albicans*. **(C)** The antagonistic efficacy photographs that indicate different inhibition zones for liquid phase of F5-F10 formulations. **(D)** Chart showing the developed inhibition zones using well diffusion method of F5-F10 formulations against (a) *Salmonella paratyphi*, (b) *Shigella spp*, (c) *Pseudomonas aeruginosa*, (d) *Escherichia coli*, (e) *Streptococcus spp*, (f) *Staphylococcus epidermidis*, (g) *Staphylococcus aureus*, (h) *Candida kruisei*, (i) *Saccharomyces cerevisiae* and (j) *Candida albicans*

By the disc diffusion method, tested synthetic films (containing CuO NPs) have suppressed the growth of some human pathogens (Fig. 3A). In case of F10 (VI) discs, the largest inhibition zones are obtained against *Saccharomyces cerevisiae* (21.89 ± 3.59), followed by *Escherichia coli* (20.12 ± 4.56), and *Pseudomonas aeruginosa* (18.36 ± 6.23) as in Table 5. However, no inhibitory zones are detected against any of tested *Gram* + ve bacteria (Fig. 3B). Secondly, via the well diffusion method, we notice that all alginate/zein/CuO NPs samples coded IV, V, and VI, as well as alginate/0.4% CuO NPs (II), suppressed the growth of all examined human pathogens (Fig. 3C). The highest hole zones are determined statistically in case of F10 (VI) against *Escherichia coli* (25.78 ± 5.96) from *Gram-ve* bacteria followed by *Saccharomyces cerevisiae*, (23.79 ± 5.69) from fungal cells and *Streptococcus spp.* (20.18 ± 3.69) from *Gram* + ve bacteria (Table 5). Additionally, the samples that did have not CuO NPs (F5, F6) show no inhibition zones by using both methods.

It can be concluded that, the antimicrobial effect is perceived due to CuO NPs, which can emit reactive oxygen species (ROS); that are responsible for damaging the bacteria’s cells [53]. Moreover, upon increasing CuO NPs concentration from 0.1 to 0.4%, w/v; the antimicrobial potential is significantly enhanced. Meanwhile, antibacterial activity of CuO towards *Gram-*negative bacteria was superior with regard to *Gram*-positive ones. The disparity in activity could be appertain to the cell membrane structure and composition. *Gram*-positive bacteria with a thicker peptidoglycan cell membranes than *Gram*-negative, make it firmer for CuO to invade it, instigating a lower antibacterial response [42].

**Fig. 4:**
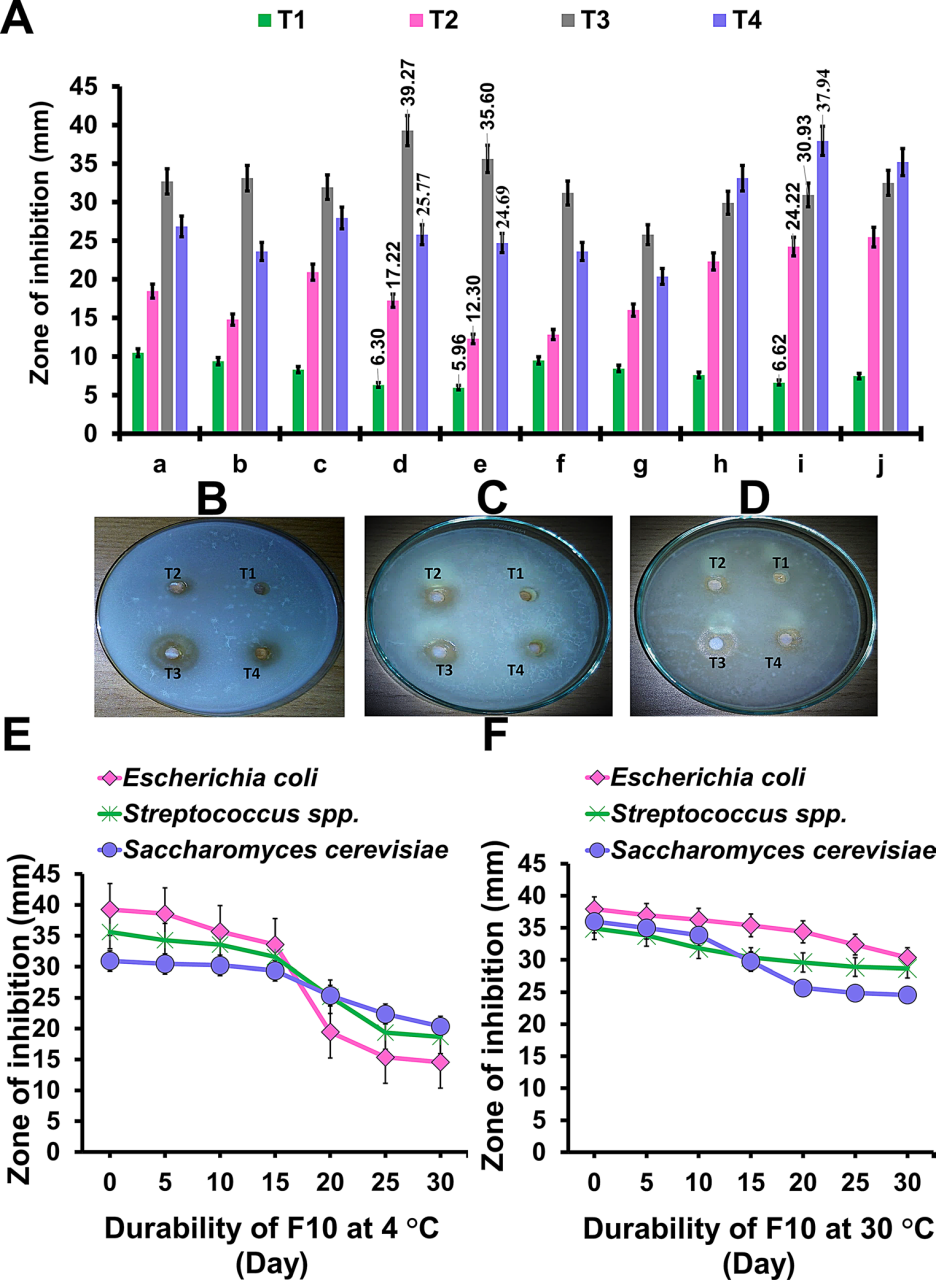
**(A)** Antagonist efficacy of dilute 0.4%w/v CuO NPs-loaded AZF (F10) formulation, (T1; 10% V/V), (T2; 30%V/V), (T3; 50% V/V) and (T4; 70% V/V) against (a) *Salmonella paratyphi*, (b) *Shigella spp.*, (c) *Pseudomonas aeruginosa*, (d) *Escherichia coli*, (e) *Streptococcus spp.*, (f) Staphylococcus epidermidis, (g) Staphylococcus aureus, (h) *Candida kruisei*, (i) Saccharomyces *cerevisiae* and (j) *Candida albicans*. These photo point to inhibition zones of dilute F10 formulation against **(B)** *Escherichia coli*, **(C)** *Streptococcus spp.*, and **(D)** *Saccharomyces cerevisiae*. The effectiveness and durability of antimicrobial activities of dilute F10 formulation (50%) stored at **(E)** 4^°^C and **(F)** 30^°^C against *Escherichia coli*, *Streptococcus spp.*, and *Saccharomyces cerevisiae*

Based on these results, we conclude that F10 (VI) formula has an effective antimicrobial activity against extremely drug-resistant human pathogens of both *Gram* -ve and *Gram* + ve strains, as well as fungal cells. To detect the effective doses, different dilutions of alginate/zein/ 0.4%w/v CuO NPs (VI) formula are prepared using Milli-Q H_2_O; such as 10, 30, 50, and 70% (v/v) that are coded as T1, T2, T3, and T4 respectively (Table 5). As can be seen in Figs. 4A-D and 30% (T2), 50% (T3), and 70% (T4) ratios respectively; are significantly increased antagonistic efficacy against all tested human pathogens. The most efficient dilution that form the largest inhibitory zone against all tested bacterial human pathogens is 50% (T3). While the most effective one in the case of fungal cells is 70% (T4). Furthermore, 10% (T1) dilution seems to have the lowest inhibitory zones against all pathogens tested. The highest hole zones are determined statistically bacterial cells; are recorded by using T3 (50%) against *Escherichia coli* (39.26 ± 3.78) followed by *Streptococcus spp.* (35.6 ± 5.12). Furthermore, T4 (70%) increased the inhibition zone against fungal cells to 37.94 ± 8.12 in the case of *Saccharomyces cerevisiae*, followed by *Candida albicans* (35.19 ± 6.09), and *Candida krusei* (33.1 ± 8.36) that can be seen in the Table 6.

**Table 6:** Anti-biofilm efficacy of different dosages of formulations against different multidrug-resistant human pathogens in-vitro

Multidrug-resistant human pathogens	Biofilm inhibition (%±SD)				
	50 (µg/mL)	100 (µg/mL)	150 (µg/mL)	200 (µg/mL)	250 (µg/mL)
*Candida krusei*	12.5 ± 6.23 ^c^	9.56 ± 2.36 ^c^	23.53 ± 17.36 ^b^	37.5 ± 5.23^b^	78.82 ± 1.28 ^a^
*Candida tropicals*	10.43 ± 1.78 ^c^	38.55 ± 8.33 ^c^	64.06 ± 4.12 ^b^	72.75 ± 3.69 ^b^	91.45 ± 4.69 ^a^
*Candida glabrata*	7.58 ± 3.59 ^c^	50.12 ± 8.12 ^c^	64.06 ± 3.56 ^b^	77.51 ± 5.09 ^b^	80.68 ± 6.45 ^a^
*Candida albicans*	25.94 ± 10.36 ^c^	22.83 ± 14.23 ^c^	39.43 ± 6.23 ^b^	47.17 ± 8.12 ^b^	85.19 ± 9.12 ^a^
*Streptococcus spp.*	12.18 ± 4.78 ^c^	22.44 ± 8.12 ^c^	30.77 ± 7.15 ^b^	37.18 ± 0.78 ^b^	73.72 ± 9.17 ^a^
*Staphylococcus epidermidis*	3.35 ± 1.09 ^c^	9.57 ± 4.12 ^c^	30.62 ± 8.12 ^b^	51.19 ± 8.94 ^b^	82.73 ± 14.23 ^a^
*Staphylococcus aureus*	7.09 ± 2.07 ^c^	18.89 ± 3.69 ^c^	51.57 ± 4.78 ^b^	58.66 ± 7.56 ^b^	75.75 ± 9.14 ^a^
*Bacillus cereus*	8.06 ± 6.82 ^c^	15.32 ± 1.85 ^c^	21.77 ± 0.78 ^b^	41.94 ± 15.36 ^b^	68.39 ± 9.18 ^a^
*Salmonella paratyphi*	44.39 ± 7.89 ^c^	47.59 ± 11.23 ^c^	71.12 ± 17.36 ^b^	75.61 ± 9.45 ^b^	87.17 ± 18.36 ^a^
*Shigella spp.*	12.55 ± 12.83 ^c^	14.71 ± 9.38 ^c^	31.60 ± 9.78 ^b^	52.81 ± 7.52 ^b^	75.84 ± 10.26 ^a^
*Pseudomonas aeruginosa*	14.53 ± 5.48 ^c^	30.41 ± 9.07 ^c^	51.01 ± 10.24 ^b^	59.46 ± 8.04 ^b^	67.91 ± 11.05 ^a^
*Escherichia coli*	11.86 ± 9.03 ^c^	31.09 ± 9.36 ^c^	36.54 ± 8.94 ^b^	44.87 ± 3.05 ^b^	91.92 ± 8.29 ^a^

Lowercase letters indicate significant differences between the biofilms formed

Similar letters indicate that there is no significant difference (*P* > 0.05). a, b, c

The antibacterial efficiency and durability of diluted F10 formula (50%) are tested against *Escherichia coli*, *Streptococcus spp.*, and *Saccharomyces cerevisiae* by packing this formula at 4 °C and 30 °C, and inhibited zones are recorded at intervals. The baseline zones of inhibition diameters (0 day) are estimated after tested composition; is prepared. According to displayed data, antimicrobial activities of this formula continued for 30 days in both cases (Fig. 4E and F). Furthermore, within 15 days, antimicrobial activities of tested formula at 4 °C and 30 °C are nearly the same against all tested strains (*p* > 0.05), suggesting that CuO NPs are released continuously from AZFs. Interestingly, the examined formula’s antimicrobial activity held steady for 30 days after being stored at 4 °C. The antimicrobial activity of the formula stored at 30 °C appear the lowest over 15-to-30-day period. During this period (15 days), the release of CuO NPs from F10 formulation (50%) exhibits a sustained antimicrobial activity, which may just protect cutaneous wounds from infection. According to this data, once this period has passed, there is no longer a risk to human health. As an outcome of this study, a sustained-release CuO NPs from AZFs (F10) at 50% concentration, results in faster wound healing which will aid to reduce the price of burns treatment by allowing for an earlier hospital discharge. As a result, this formula is expected to become a valuable wound and burns therapy prefect tool in the future.

### In vitro cell viability

The cytotoxicity of CuO NPs loaded AFs (F7), AZFs (F10), as well as CuO NPs on HBF4 cells over the range (0-0.8%) are tested (Fig. 2D). Both films exhibited higher safety on the viability of HBF4 cells than free CuO NPs. The estimated IC_50_ values are 1.002 ± 0.05%, 1.487 ± 0.06%, and 1.933 ± 0.05% for free CuO NPs, CuO NPs AFs (F7), and CuO NPs-loaded AZFs (F10); respectively. Additionally, the safe doses (EC_100_) are 0.071 ± 0.001%, 0.097 ± 0.007%, and 0.121 ± 0.001%; respectively. This indicates the highest IC_50_ and EC_100_ values of CuO NPs-loaded AZFs (F10), compared to AFs and free-CuO NPs.

Zein proposes extra potential benefits as a cyto-compatible protein for proliferation of NIH3T3 cells as well as HL-7702 cells [9]. It is noticed that cell viability is dependent on the concentration of loaded-CuO NPs, where cell viability decreases with increasing concentration. Lipid peroxidation and oxidative stress have been previously reported to be one of the toxicity mechanisms related to CuO NPs exposure [54]. It is also noticed that cell viability of 0.4% w/v loaded AZFs (F10) ranged from 60 to 89%. This may be attributed to its green nature synthesis, where CuO NPs are coated with capping and stabilizing agents from the plant extract which enhancing its biocompatibility. The same is observed when CuO NPs are previously coated with biopolymers, such as hyaluronic acid. The obtained results for cell viability show that ions like Cu^2+^ can be deliberated as safe ions for biomaterial application specially at low doses [55]. 

### In vivo wound healing potency

#### Wound healing appraisal

Alginate and zein could have the capability to synergistically accelerate the diabetic wound healing process in the rat model. Both polymers can act as reservoirs for active agents, biocompatible, and biodegradable. Alginate has a hemostatic property, while zein has antimicrobial and antioxidant properties. The diabetic wound healing potential of the biopolymers was tested on circular wounds on the dorsal area of diabetic rats [6, 14, 56].

Principally, copper, has a crucial starring role in wound restoration by stimulating angiogenesis, and enhancing cell migration with proven wide spectrum bactericidal effect claiming for copper oxides application to be convenient, but at the same time risky [21, 57, 58]. In this regard, the incorporation of CuO NPs into AZF is intended to control the release of CuO NPs, due to the presence of hydrophobic polymer like zein and CaCl_2_ as a crosslinker. Such a retardation in the released amount would reduces the cytotoxicity and enhances the healing process.

**Fig. 5:**
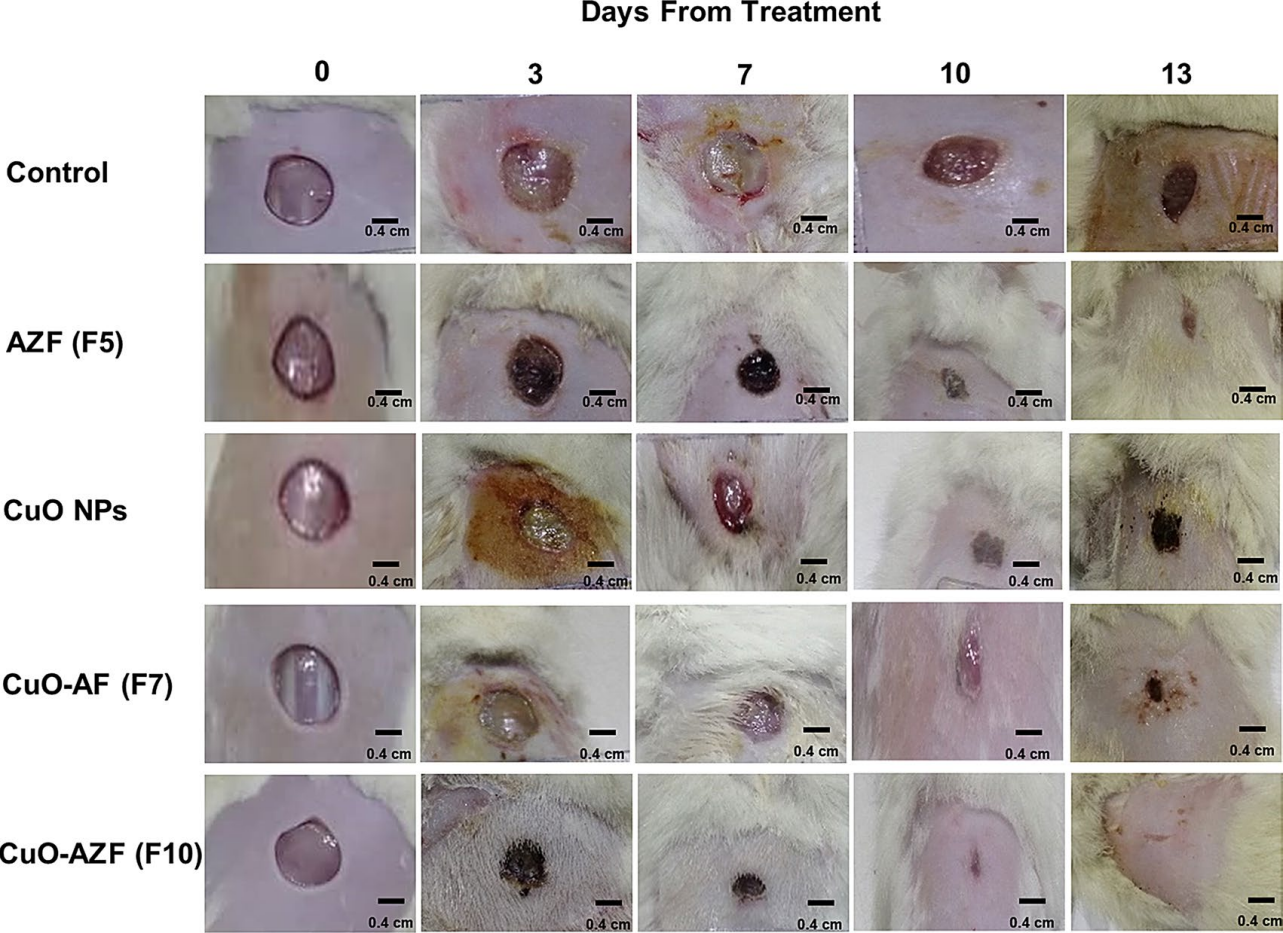
Photographic images of in-vivo excision wound area contraction rate vs. day of observation using dorsal of rats. The scale bar represents 0.4 cm

Photographs of the wound spots covered with films and the evaluated mean wound contraction rate are depicted in Fig. 5 and Table 7; respectively. It is visually clear that the untreated group deteriorated with unpaired healing, while treated groups show good healing. However, the application of CuO-loaded AZF (F10) has the superb most significant effect than other groups. This implanted by a reduction of WC50 values of CuO powder, unloaded F6, F7 and F10-treated groups (10.6, 7.0, 7.5, and 4.4 days; respectively) are lower than those observed in the control group (11.7 days) (Table 7). Both plain and loaded AZFs (F7, F10) show the most protuberant improvement, compared to control and other groups with 88.5 ± 1.9 and 98.7 ± 2.4 WC%; respectively after 13 days, executing the role of zein in enhancing wound healing process and augmenting the CuO NPs role in the activation of both fibroblast and collagen fibers in injured site, promoting wound tissues regeneration [59, 60] (see Table 8).

**Table 7:** Effect of the application of CuO nps, blank AZF (F6), 0.4%w/v CuO-AF (F7), and 0.4%w/v CuO-AZF (F10) films on excision wound area contraction and half closure time (values are mean ± sd, *n* = 6 observations in each group)

Day	Wound contraction (%)				
	Control	AZF (F6)	CuO NPs	CuO-AF (F7)	CuO-AZF (F10)
3	7.3 ± 0.15	22.7 ± 2.2	10.1 ± 2.6	17.8 ± 2.1	37.2 ± 1.5
7	10.2 ± 1.7	50.1 ± 2.4	30.5 ± 1.9	47.3 ± 1.6	72.6 ± 1.2
10	35.4 ± 2.1	76.3 ± 2.9	45.1 ± 2.8	64.2 ± 2.5	82.9 ± 0.9
13	62.3 ± 1.8	88.5 ± 1.9	65.0 ± 2.4	79.1 ± 2.1	98.7 ± 2.4
*WC_50_ (day)	11.7	7.0	10.6	7.5	4.4

Abbreviations: *WC_50_: half closure time of wound contraction in days

Notes: Values are significant (#) at *P* < 0.05 as compared to the untreated group (control)

**Table 8:** The influence of the application of CuO nps, blank AZF (F6), 0.4%w/v CuO-AF (F7), and 0.4%w/v CuO-AZF (F10) films on the re-epithelization and granulation of the wound, immuno-histological parameters (values are mean ± sd, *n* = 6 observations in each group)

Immuno-histological parameter/HPF	Control	AZF (F6)	CuO NPs	CuO-AF (F7)	CuO-AZF (F10)
Collagen Fibers	1.2 ± 0.7	93 ± 4.2	6.8 ± 4.7	88 ± 2.9	75 ± 4.2
Blood Vessels	32 ± 2.4	3 ± 1.6	66 ± 1.8	7 ± 3.3	23 ± 3.6
Myofibroblasts	43 ± 3.1	7 ± 2.3	48 ± 8.3	10 ± 2.6	30 ± 6.1
Lymphocytes	82 ± 2.6	2 ± 1.6	64 ± 2.8	40.1 ± 2.1	47 ± 5.3

#### Histological examination

**Fig. 6:**
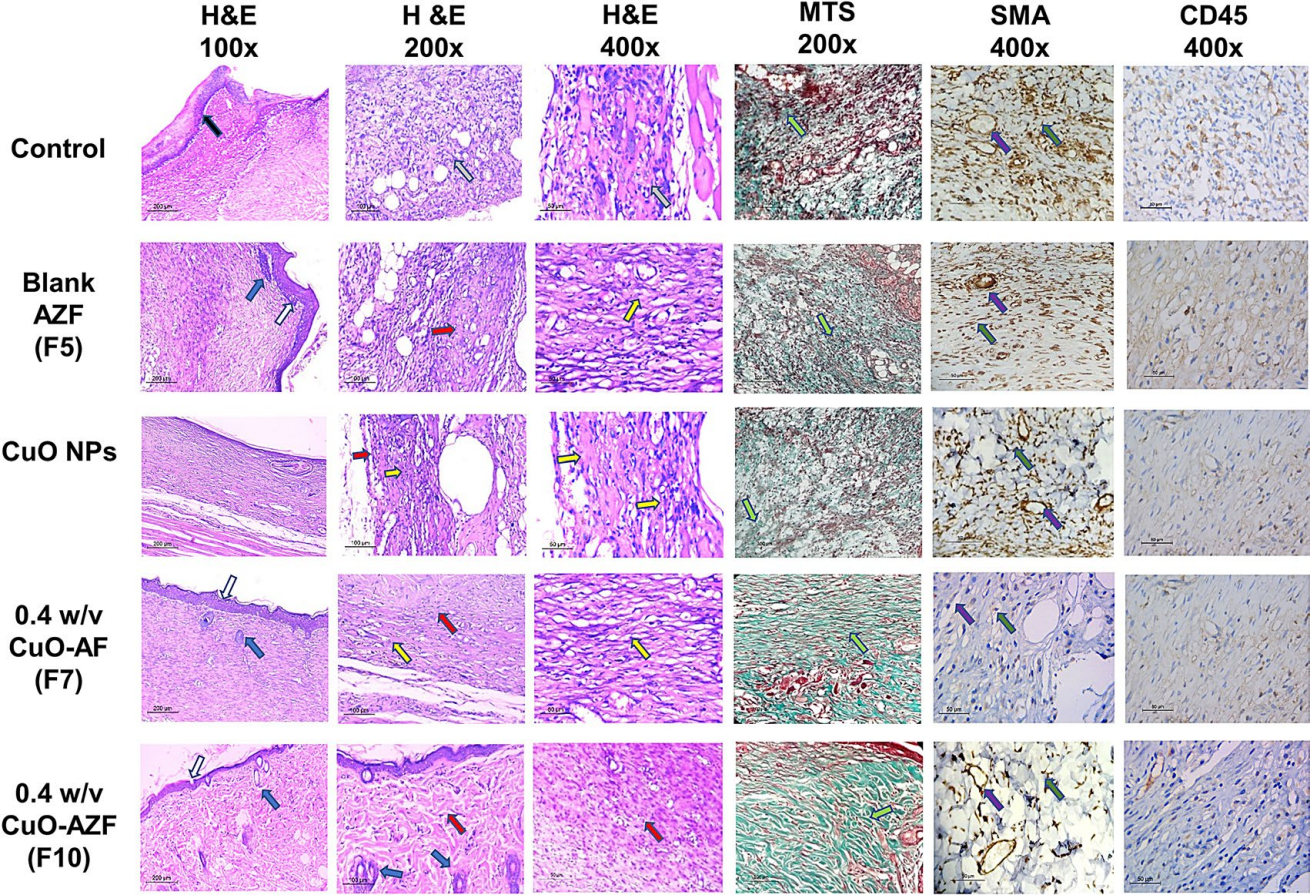
Histological examination of H&E and MTS-stained sections of wounds, and immunohistochemical (IHC) staining of excised skin sections using SMA and CD45

Histological examination of all skin sections visualizes the significance of each of the treatments. It enables tracking the healing process advancement (phases of wound healing, skin adnexal regeneration, epidermal re-epithelialization, as well as the amount of inflammatory infiltrate). Control group H&E-stained wounds (Fig. 6) showed an improper wound healing. The ulceration of the epidermis was observed and shielded by fibrinoid exudate entangling inflammatory cells (black arrow) (H&E, x100) with heavy inflammatory infiltrate underlying tissues (green arrow) (H&E, x200, x400. Unloaded AZFs (F6)-treated groups depict the early remodeling phase healing. The epidermis showed epithelialization with few skins’ adnexal regeneration, mild fibrous tissue deposition and a moderate amount of granulation tissue. The wound sections from CuO NPs-treated group were in the inflammatory as sections revealed extensive inflammation and granulation tissue (yellow arrow) with residual epidermal ulceration (black arrow) and mild fibrosis (red arrow) (H&E, x100) with increasing number of inflammatory cells (H&E, x200, x400). Both CuO NPs loaded AFs (F7) and CuO NPs loaded AZFs (F10)-treated groups show similar findings, where the sections demonstrate the remodeling phase of the healing process, an intact fully thickened keratinized epidermal epithelialization, thickness (purple arrow) and the complete absence of any inflammatory infiltrate. However, the histological outcome in CuO NPs loaded AZFs (F10)-treated group illustrates a significant enhancement in skin adnexal regeneration (blue arrow) with extensive fibrous tissue deposition (red arrow) (M, H&E, x100) and significant decrease in residual granulation tissue (yellow arrow) and few residual inflammatory cells (H&E, x200, x400) compared to CuO NPs loaded AFs (F7)-treated group. The histological outcome confirms the obtained in-vivo results.

#### Immunohistochemical (IHC) staining and interpretation

Skin specimens from the group treated with F6 show epidermal re-epithelialization 47 ± 4.3 μm with the regeneration of the appendages ≥ 5/wound area (score 3). The dermis is founded mostly of both collagen fibers and fibrous tissue 93 ± 4.2% (remodeling phase). Minutest granulation tissue is noticed and formed of a few fresh blood vessels 3 ± 1.6/HPF, few myofibroblasts 7 ± 2.3/HPF and few lymphocytes 2 ± 1.6/HPF, (Fig. 6). Skin specimens from the untreated control group show extensive crusted ulcerative epidermis (43 ± 1.9 μm), deprived of skin appendages. The dermis rendered in the inflammatory phase with fibrous tissue (1.2 ± 0.7/HPF). A neovascularization of 32 ± 2.4/HPF was recognize with myofibroblasts of 43 ± 3.1/HPF, and lymphocytes (82 ± 2.6/HPF). Skin specimens from the group treated with F7 depicted a remodeling phase of healing with normal epidermal epithelialization, thickness 45 ± 3.5 μm and limited skin adenexal regeneration (score 2) with extensive fibrous tissue deposition 88 ± 2.9/HPF and mild residual granulation tissue formed of myofibroblasts 10 ± 2.6/HPF and newly formed blood vessels 7 ± 3.3/HPF. Group treated with F6 depicts an early remodeling phase of healing showing an epithelialization of the epidermis with rare skin adnexal regeneration (score 1) and mild fibrous tissue deposition 75 ± 4.2/HPF with a moderate amount of granulation tissue, myofibroblasts 30 ± 6.1/HPF, blood vessels 23 ± 3.6 and lymphocytes 47 ± 5.3/HPF. Group treated with CuO NPs depicts diminutive amounts of fibrous tissue (6.8 ± 4.7/HPF), myofibroblasts 48 ± 8.3/HPF with 66 ± 1.8/HPF of newly formed blood vessels, and 64 ± 2.8/HPF of lymphocytes (Fig. 6).

## Conclusions

A series of alginate or alginate/zein films with different CuO NPs content were customized and utilized as wound dressings for injured skin in diabetic rat model. The film dressings were tunable in terms of their surface structure and mechanical properties by changing the cross-linking method and CuO NPs content. The films exhibited good cytocompatibility to HBF4 cells. Furthermore, alginate/zein film containing 0.4% CuO NPs content provides useful insights to the tailoring of wound dressings which could promote wound contraction rates with epidermal epithelialization and skin adnexal regeneration confirming their excellency as wound dressings candidates for skin tissue engineering.

## Data Availability

The datasets used and analyzed during the current study are available from the corresponding author on reasonable request.
